# Is paravertebral block the optimal analgesic modality for non-mastectomy breast surgery?

**DOI:** 10.3325/cmj.2025.66.238

**Published:** 2025-06

**Authors:** Lora Kukić, Miroslav Župčić, Nataša Višković Filipčić, Nina Sulen, Viktor Duzel, Dinko Tonković

**Affiliations:** 1 Department of Anesthesiology and Intensive Care Medicine, Rijeka University Hospital Center, Rijeka, Croatia; 2 Department of Clinical Sciences II, Faculty of Health Studies, University of Rijeka, Rijeka Croatia *miroslav.zupcic@uniri.hr*; 3 Special Hospital Agram, Zagreb, Croatia; 4 Department of Anesthesiology, Resuscitation and Intensive Care Medicine, Zadar General Hospital, Zadar, Croatia; 5 Department of Health Studies, University of Zadar, Zadar, Croatia; 6 Northern Hospital Epping, Victoria, Melbourne, Australia; 7 Department of Anesthesiology, Resuscitation and Intensive Care Medicine, University of Zagreb School of Medicine, Zagreb, Croatia; 8 Department of Anesthesiology, Reanimatology and Intensive Care Medicine, Zagreb University Hospital Center, Zagreb, Croatia

Thoracic paravertebral block (TPVB) is a well-established regional anesthesia technique used for postoperative pain management after breast surgery, including both mastectomy and non-mastectomy procedures (1). Non-mastectomy breast surgeries, such as lumpectomy, breast reconstruction, breast reduction, mastopexy, and implant/expander removal or placement, can also cause considerable postoperative pain. Effective pain management is crucial for ensuring patient comfort, facilitating early mobilization, and preventing the development of chronic post-surgical pain (2,3). This is particularly important in the context of increasingly common ambulatory surgery, where 12% of patients experience chronic postsurgical pain and 9% report moderate to severe pain (2). Traditional analgesic options for breast surgery include systemic opioids, local anesthetic infiltration, and regional techniques such as paravertebral, pectoralis-II, serratus plane, or erector spinae blocks, whereas TPVB stands out for its high success rate and a low risk of serious complications such as pneumothorax (0.5%) (4). While PVB has demonstrated efficacy and safety in mastectomy procedures, its role and potential advantages over other analgesic methods in non-mastectomy surgeries require further investigation (1-5).

A recent randomized controlled trial by Gabriel et al (6) directly compared the efficacy of PVB and pectoralis-II blocks in 119 patients undergoing non-mastectomy breast surgeries. The primary outcomes of the study were pain scores and opioid consumption. Pain scores were assessed using an 11-point numeric rating scale (0 to 10, where 0 indicates no pain and 10 indicates the worst pain imaginable). Opioid consumption was assessed by converting intravenous fentanyl, intravenous hydromorphone, and oral oxycodone to intravenous morphine equivalents. PVB was associated with lower pain scores in the recovery room and reduced opioid consumption in both the operating and recovery rooms compared with pectoralis-II blocks. Specifically, participants who received PVB reported a median pain score of 1.3 in the recovery room, compared with 3.3 in those who received pectoralis-II blocks. Additionally, the PVB group had a significantly lower total opioid consumption (intravenous morphine equivalents) in the operating room and recovery room combined (median 10.0 mg) compared with the pectoralis-II group (median 17.5 mg) (6). These findings suggest that PVB may offer superior analgesia in non-mastectomy procedures by providing better pain control and reducing the need for opioids.

In 2021, Gabriel et al conducted a randomized controlled trial to compare the effectiveness of serratus plane block (SPB) and PVB in managing postoperative pain in 100 patients undergoing non-mastectomy breast surgery (7). The primary outcomes were pain scores in the recovery room and opioid consumption in both the operating and recovery room. The results showed that PVB provided superior analgesia, with significantly lower pain scores in the recovery room (median numeric rating scale score of 0 vs 4). While the difference did not reach statistical significance, opioid use was reduced in the PVB group (median morphine equivalents of 10 mg vs 14 mg). Interestingly, SPB had a shorter procedural time than PVB (1.7 min/side vs 4.3 min/side), while the pain experienced during the block procedure was similar between the groups (7). These findings indicate that, while SPB may be quicker to perform, PVB provides more effective pain relief after non-mastectomy breast surgery, with both procedures being similarly painful.

While PVB offers effective pain control and reduced opioid consumption, it may lead to potential complications. Pneumothorax, a rare but serious complication, occurs due to the proximity of the pleura to the paravertebral space. In a study of 367 patients who underwent PVB, 4.6% of participants developed hypotension, 3.8% vascular puncture, 1.1% pleural puncture, and 0.5% pneumothorax. Also, PVB may transform into an epidural block or even total spinal anesthesia. Other potential complications include vascular puncture (3.8%), nerve injury, and local anesthetic toxicity, while the overall failure rate has been 10.1% (8). The incidence of complications varies significantly, however, depending on the experience of the provider, the patient's anatomy, and the specific technique used (9).

The optimal dose and volume of local anesthetic for PVB depend on the type of surgery, patient characteristics, and the desired duration of analgesia. In general, lower concentrations and volumes are associated with a lower risk of complications, while higher concentrations and volumes may provide longer-lasting analgesia. The choice between single-shot and multiple-shot PVB also depends on the specific surgical procedure and the desired duration of analgesia. Single-shot PVB is typically sufficient for shorter procedures, while multiple-shot or continuous PVB may be preferable for longer procedures or when prolonged analgesia is desired (10).

As mentioned, the choice of local anesthetic for PVB can significantly affect hemodynamic stability, a crucial aspect of patient safety during surgery. Župčić et al (3) investigated the hemodynamic effects of different local anesthetics used for PVB in patients undergoing quadrantectomy, a non-mastectomy breast surgery. The study compared the use of 0.5% levobupivacaine alone vs a combination of 0.5% levobupivacaine and 2% lidocaine for PVB. The primary outcome was the time to block onset, and the secondary outcomes included hemodynamic variables such as stroke volume variation (SVV), mean arterial pressure, heart rate, and cardiac output. The study also assessed the duration of analgesia and postoperative pain using a visual analogue scale (VAS). The results indicated that levobupivacaine alone provided a longer time to block the onset but also reduced hemodynamic disturbances and prolonged the analgesic effect compared with the combination. Specifically, the combination of levobupivacaine and lidocaine resulted in a shorter time to block onset (mean onset 14 minutes) but significantly increased SVV values over the 60 minutes of monitoring. Furthermore, the patients who received the combination experienced a shorter mean duration of analgesia (105 minutes) and more episodes of hypotension (17.5%), and received more intraoperative crystalloids (mean volume: 550 mL) (11). These findings highlight the importance of selecting appropriate local anesthetics for PVB to minimize hemodynamic disturbances and optimize postoperative analgesia. The use of levobupivacaine alone for PVB in non-mastectomy breast surgery may provide a more favorable hemodynamic profile and longer-lasting analgesia compared with the combination with lidocaine.

In conclusion, PVB appears to be a promising analgesic option for non-mastectomy breast surgeries, potentially offering benefits in terms of lower pain scores and reduced opioid consumption. However, careful consideration of potential risks and benefits, appropriate technique, local anesthetic selection, and the expertise of the anesthesia provider are crucial for optimizing patient outcomes. The available evidence suggests that PVB may be superior to other analgesic modalities in non-mastectomy breast surgeries, but further research is warranted to corroborate these findings and optimize the use of PVB in these procedures.
